# Supplementary material for: Gene Expression Profiles Deciphering the Pathways of Coronatine Alleviating Water Stress in Rice (Oryza sativa L.) Cultivar Nipponbare (Japonica)
Source: Int J Mol Sci. 2019 May 23;20(10):2543. doi: 10.3390/ijms20102543 (PMC6567010; doi:10.3390/ijms20102543)
Supplement: Supplementary file 1 [file ijms-20-02543-s001.pdf]

Table S1. Gene-specific primers for RT-qPCR analysis.

Gene Name		Primer	Product Size (bp)
Os01g0298400	Forward	5’ -CACGAGCGTTGGGATCAGT-3’	147
	Reverse	5’ -AAATTACTCCACCAGCAGTTGTCTC-3’	
Os01g0597800	Forward	5’ -ATGATCGACCCAAAGAAACG-3’	92
	Reverse	5’ -CTGTTGAACACCTCGCCATA-3’	
Os01g0826400	Forward	5’ -GACATGTCGGAGCATTCCTT-3’	99
	Reverse	5’ -GGAGCCAACTCTGATCTCGT-3’	
Os02g0278400	Forward	5’ -GATCAGCACCGCAAACTA-3’	131
	Reverse	5’ -AAACCCTAGCCGTCACTT-3’	
Os02g0426800	Forward	5’ -TTCTTCGTCCTCGTCTTCGT-3’	104
	Reverse	5’ -CTCCTGTGCTTCCTGACCTC-3’	
Os02g0572200	Forward	5’ -ATCCACGCGGGTACTACAAG-3’	179
	Reverse	5’ -GTCGTAGGTGACGATGAGCA-3’	
Os04g0447700	Forward	5’ -GCAGAACTGGATAGGCGAAG-3’	144
	Reverse	5’ -TTGTAGCTCTTGGGGATTGG-3’	
Os04g0578000	Forward	5’ -GTTGCTGGAGGTCAGGAATAAAG-3’	148
	Reverse	5’ -GACGGGTTGGTGATGAGGA-3’	
Os08g0520500	Forward	5’ -TTTGCATGTAGCTGGTGGAC-3’	179
	Reverse	5’ -TCGTAGGACCCAACACAACA-3’	
Os09g0479800	Forward	5’ -CAACTCCAGCGAGTGCATTA-3’	128
	Reverse	5’ -TTGATTTCTTGGCTGCTGTG-3’	
actin	Forward	5’ -TTATGGTTGGGATGGGACA-3’	292
	Reverse	5’ -AGCACGGCTTGAATAGCG-3’	
